# Simpler intake estimation using direct observation in small ruminants: grouping bites by plant structure and morphology

**DOI:** 10.1186/s13104-018-3570-8

**Published:** 2018-07-11

**Authors:** P. G. González-Pech, J. F. J. Torres-Acosta, C. A. Sandoval-Castro

**Affiliations:** 1 Facultad de Medicina Veterinaria y Zootecnia - Universidad Autónoma de Yucatán, Km 15.5 Carretera Mérida-Xmatkuil, 97315 Mérida, Yucatán Mexico; 2 Centro Multidisciplinario de Educación Ciencia y Cultura SCP, Calle 35C No 43 Fracc. Colonial Buenavista, 97215 Mérida, Yucatán Mexico

**Keywords:** Intake estimation, Sheep, Goat, Browsing, Bite monitoring, Bite category, Behaviour

## Abstract

**Objective:**

To validate the estimation of dry matter intake (DMI) obtained from bite categories (BC) and weight for every plant species (method 1: M1) vs. an alternative method (method 2: M2) grouping plants based on structure and leaf morphology. A dataset containing 80,813 bites and 33 plant species obtained by M1 for sheep and goats browsing a tropical forest was used. Plant species and their respective bite weight were regrouped according to M2. BC weights within each morphological group were compared using ANOVA and post hoc Tukey’s honest significant difference comparisons. DMI was estimated for sheep, goats and DMI obtained with both approaches was compared using the t-test, Pearson correlation and orthogonal regression analyses.

**Results:**

Dry matter intake estimations were: M1 = 369 ± 153 vs. M2 = 425 ± 161 gDM for sheep and M1 = 567 ± 190 vs. M2 = 681 ± 203 gDM for goats. DMI estimations by M1 and M2 were similar and strongly correlated. Orthogonal regression showed both procedures yielded a similar DMI estimation (P < 0.001). M2 reduces the amount of work required to estimate DMI in heterogeneous vegetation without reducing accuracy. M2 reduced the time required and made simpler to include data from larger number of animals/replicates.

## Introduction

The improved direct observation method to the continuous record of feeding behavior developed by Agreil and Meuret [1], and validated by Bonnet et al. [2], has been adapted to study the grazing behaviour of sheep and goats in tropical heterogeneous vegetation (method 1: M1) [3, 4]. However, to implement M1 requires a large investment of time to train the observers to: (i) differentiate several plant species at their different phenological stages, (ii) identify bite categories (BC) amongst the different plant species, and (iii) perform the hand plucking method to obtain the BC weight. Consequently, grouping few groups of plants according to their similar morphology could save time and efforts devoted on the steps i, ii and iii. Such simplified method, might favour its use in the tropics and other types of diverse and heterogeneous ecosystems. Using M1, under the conditions of tropical vegetation, it was found that several plant species share similar structures and leaf morphology [3]. Thus, they might also share a similar weight for each BC. We hypothesized that identification of the BC for every plant species (M1) can be used to group plants by their structure and leaf morphology to estimate dry matter intake (DMI) (method 2: M2). Therefore, M2 would require less work to estimate the DMI of browsing sheep and goats. Therefore, the objective of the present study was to compare the estimation of the DMI obtained with both methods (M1 and M2) and to validate the M2 approach as a simpler direct observation method compared with M1.

## Main text

### Methods

#### Bite categories by the direct observation method (M1) dataset

A dataset from a previous experiment using M1 with sheep and goats (34 ± 3 kg live weight, adults, non-pregnant) browsing in 112 ha of deciduous tropical forest in México was used [4]. It contained 80,813 observed bites according to five types of plant parts harvested (leaves only, leaves + stem, branch with many leaves + stems, vines and grass), each with six different bite sizes (1–3, 3–5, 6–10, 11–15 and 16–20 cm). It included 33 plant species and the weight of their respective bites, resulting in 212 BC and their respective weight via the hand plucking method. The range of weight (g dry matter [DM]) for BC were from 0.031 to 0.439 for grasses, 0.004–1.800 for herbaceous, 0.005–2.050 for woody plants, 0.068–1.585 for woody bipinnate leaves, and 0.022–0.654 for vines plants.

#### Bite categories by morphological grouping (M2) dataset

Morphological grouping (M2) was obtained from the above M1 dataset by grouping BC into nine morphological groups: (a) grasses (b) small herbaceous plants (< 20 cm height), (c) big herbaceous plants (> 20 cm height), (d) small woody shrubs (leaves < 3 cm width), (f) big woody shrubs (leaves > 3 cm width), (g) small bipinnate leaves (individual leaves are short < 10 mm long, and rounded), (h) medium bipinnate leaves (individual leaves are elongated > 10 mm long and thin < 2 mm wide), (i) large bipinnate leaves (individual leaves are elongated and rounded), and (j) vines. Then, DMI was obtained by two procedures: M1, the accumulated sum of weight data from all BC performed on every plant species consumed by animals and M2, the accumulative sum of the product of the mean BC weight and total bites grouped into morphological groups.

#### Statistical analysis

An assessment of the similarity amongst BC from the species contained on each BC cell (for M2) was carried out. As BC by morphological groups had variable number of species the Kolmogorov Smirnov test was use to assess the normality of BC weight of each plant species. Then, parametric (ANOVA or t-test) or non-parametric tests (Kruskal–Wallis) were used to compare the BC weight of different plant species grouped into the same BC for each morphological group. Morphological grouping dataset arising from different species were similar and allowed their used to validate M2 BC (data not shown).

The resulting dataset of BC (M2) had normal distribution, therefore, the BC by morphological group data set was analysed to validate that a larger bite (BC) represented a larger mass using ANOVA and post hoc Tukey’s honest significant difference comparisons [5].

Finally, estimation of DMI obtained with the M1 and M2 approaches were compared using the t-test, Pearson correlation and orthogonal regression analyses [6]. Orthogonal regression analyses was used as both axis (methods) *x* and *y* have error. A value of P < 0.05 was considered statistically significant.

### Results and discussion

A total of 63 BC to be used in M2 were obtained from the nine morphological groups. Ten of these BC represented a single plant species and 53 BC included two to seven plant species. Nine BC containing two or more plant species had a similar weight for each plant species within the group. For the remaining 44 BC, differences were found regarding the weight of bites amongst the different plant species but they were nevertheless integrated into M2 categories because the working hypothesis was that averaging the weight of the species according to morphological groups yields similar estimations of the DMI. Thus, the mean weight of the grouped BC in M2 ranged from 0.088 to 1.582 g DM for materials including leaves only from foliage and from 0.011 to 1.633 g DM for materials composed of leaves + stems (Table 1).

**Table 1:** Weight (mg DM) of bite categories grouped by morphological group (M2) (mean ± standard error)

Morphological	Bite categories: leaves alone					Bite categories: leaves + stems				
Group^1^	< 1 cm	3–5 cm	6–10 cm	11–15 cm	16–20 cm	< 1 cm	3–5 cm	6–10 cm	11–15 cm	16–20 cm
Grass	33 ± 1^a^ n = 30	122 ± 3^b^ n = 30	256 ± 10^c^ n = 30	396 ± 11^d^ n = 20	404 ± 6^d^ n = 10	–	–	–	–	–
Small herbaceous	8 ± 1^a^ n = 20	19 ± 1^ab^ n = 30	53 ± 8^c^ n = 20	–	–	11 ± 0.5^a^ n = 20	280 ± 1^b^ n = 10	32 ± 2^b^ n = 10	–	–
Big herbaceous	25 ± 4^a^ n = 85	78 ± 8^ab^ n = 75	137 ± 19^c^ n = 60	385 ± 58^c^ n = 40	889 ± 72^d^ n = 20	50 ± 8^a^ n = 60	115 ± 11^ab^ n = 70	176 ± 18^b^ n = 40	461 ± 65^c^ n = 50	1214 ± 115^d^ n = 30
Small woody shrubs	109 ± 1^a^ n = 30	23 ± 3^a^ n = 30	102 ± 4^b^ n = 30	167 ± 4^c^ n = 30	–	22 ± 3^a^ n = 30	85 ± 10^b^ n = 30	188 ± 5^c^ n = 25	301 ± 11^d^ n = 30	684 ± 116^e^ n = 10
Big woody shrubs	64 ± 5^a^ n = 39	105 ± 5^ab^ n = 35	222 ± 19^b^ n = 35	400 ± 44^c^ n = 30	750 ± 141^d^ n = 15	134 ± 9^a^ n = 29	221 ± 6^a^ n = 25	585 ± 8^b^ n = 15	1350 ± 8^c^ n = 15	1633 ± 166^c^ n = 20
Small bipinnate	110 ± 8^a^ n = 20	145 ± 12^ab^ n = 20	184 ± 17^b^ n = 20	–	–	–	–	–	–	–
Medium bipinnate	99 ± 7^a^ n = 20	150 ± 14^b^ n = 20	266 ± 3^c^ n = 10	755 ± 10^d^ n = 10	922 ± 21^e^ n = 10	–	–	–	–	–
Big bipinnate	76 ± 3^a^ n = 30	98 ± 4^a^ n = 30	296 ± 5^b^ n = 30	1306 ± 31^c^ n = 30	1582 ± 31^d^ n = 20	–	–	–	–	–
Vines	40 ± 4^a^ n = 39	111 ± 7^b^ n = 39	159 ± 5^c^ n = 39	209 ± 7^d^ n = 29	362 ± 21^e^ n = 10	71 ± 7^a^ n = 39	195 ± 11^b^ n = 29	317 ± 11^c^ n = 29	0.412 ± 30^d^ n = 10	654 ± 20^e^ n = 10

^a–e^ For each category (leaves alone and leaves + stems), the mean values within a row with a different superscript differ (Tukey’s post hoc honest significant difference test, P < 0.05)

^1^ Species listing for each group: Grass: *Chloris inflata, Eragrostis ciliaris var. ciliaris, Eragrostis amabilis.* Herbaceous small: *Sida acuta, Blechum pyramidatum, Tetramerium nervosum.* Herbaceous big: *Althernatera flavescens, Cnidoscolus aconitifolius, Bourreria pulchra, Parthenium hysterophorus, Solanum trydanum, Viguiera dentata, Waltheria indica, Morinda royoc.* Woddy small: *Dyospirus anisandra, Gymnopodium floribundum, Randia aculeata.* Woddy big: *Bunchosia swartziana, Cordia alliodora, neomillspaughia emarginata, Piscida piscipula.* Bipinnate small: *Senegalia gaumeri, Mimosa bahamensis.* Bipinnate medium: *Acacia collinsii, Acacia pennatula.* Bipinnate big: *Leucaena leucocephala, Lysiloma latisiliquum, Caesalpinia gaumeri.* Vines: *Bahuinia divaricata, Ipomea crinicalyx, Ipomea nill, Cardiospermum alicacabum*

^2^ The symbol “–” indicates that none of the species fall within this bite category group

**Fig. 1:**
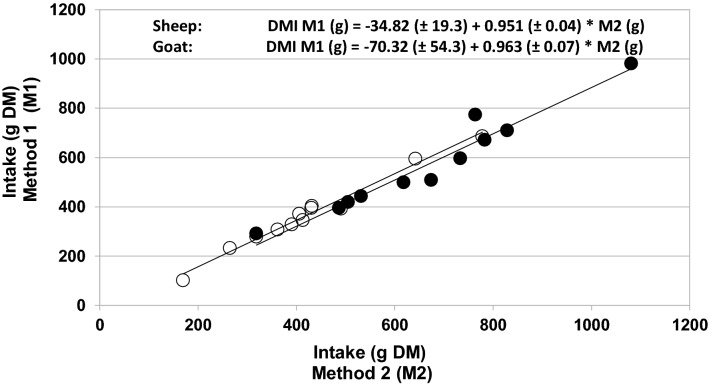
Relationship between the dry matter intake (DMI) of sheep (open circle) and goats (filled circle) estimated by the plant species method (M1) and morphological group method (M2)

Similar DMI were obtained when using either the M1 or M2 approach. For sheep, the estimated DMI by the M1 and M2 approaches were 369 ± 153 and 425 ± 161 g DM, respectively (P > 0.05). For goats, the estimations of the DMI with M1 and M2 were 567 ± 190 and 680 ± 203 g DM, respectively (P > 0.05). The Pearson correlation coefficients for the DMI obtained with M1 and M2 were 0.96, 0.99 and 0.92 for the pooled, sheep-only and goat-only data, respectively. In all cases, orthogonal regression analysis showed that the slope was not different from 1 and the intercept was no different from 0 indicating that the two procedures yielded a similar estimation of the DMI (P < 0.001) **(**Fig. 1).

To the best of our knowledge, there has been no published report of the estimation of the DMI of small ruminants (sheep and goats) using groups of plant species sharing similar morphology by bite features (morphology of bite, size or weight) as was performed here using the M2 approach. By grouping the weight of 212 BC (M1) of different plant species by their structure and leaf morphology, the original large number of plant-bite combinations for the plant diverse and heterogenous grazing/browsing tropical area [3] was reduced to only 63 (M2). The selected range span while constructing BC sizes (5 cm) represented a compromise to reduce the number of BC combinations while maintaining the lowest possible bias. As a result, the estimation of the DMI with the M1 and M2 approaches was equally reliable for sheep and goats (Fig. 1). Thus, any possible under or over estimation in the BC mean weight in M2 compared with the original BC (M1) was weighted by the mixture of plants selected and BC performed by animals during grazing/browsing.

The use of M2 simplified the data collection process to obtain the DMI by grouping the array of plant species according to their structure and leaf form to obtain morphological groups and their respective weight of BC without the need of botanical identification for every plant species and their respective weight for each BC. Thus, time can be saved sampling (via the hand plucking method) only plants that represent the morphological group and not all the plant species available. Identifying the weight of BC of morphological groups for a given vegetation with M2 can also be useful to obtain an indirect estimation of the potential biomass available to animals. Monitoring the availability of morphological groups probably could help to identify when the biomass has become insufficient for the number of animals grazing on heterogeneous vegetation. The monitoring of morphological groups available on a given vegetation is a similar approach to the GRENOUILLE method [7] that identifies the absence of big weight bites on a grazing area as an indicator to move animals to another paddock. Furthermore, the use of M2 during the continuous bite monitoring of feeding behaviour by direct observation [1, 4] can save time invested to training observers in the identification of plants consumed by the experimental animals.

## Strengths

The validation of the M2 approach allows researchers to select either the M1 or M2 approach according to the objective of a given study. For example, studies only assessing the estimation of the DMI and total nutrient intake could use the M2 approach, while experiments to determine a detailed knowledge resource selection, diet composition and quality should employ the M1 approach. However, the M2 approach helps to reduce the time invested in training observers in plant species identification as well as in BC weight estimation. Thus, M2 opens the possibility of building larger and more robust experimental studies by facilitating a larger number of replicates. Moreover, the use of M2 brings closer the possibility of using video recording systems to the estimation of the DMI on free-grazing animals by monitoring their behaviour. This would be possible because the identification of every plant species would no longer be needed. This will allow for larger and more frequent studies on ruminant grazing ecology and nutrition. Video recording is limited to studies of the feeding behaviour of housed ruminants [8, 9], which are useful but yield limited information that can be extrapolated to free range animal behaviour.

## Conclusions

Both the M1 and M2 approaches provided similar estimations of the DMI in sheep and goats feeding on a tropical forest. Using BC groups based on similarity of plant morphology (M2) reduces the amount of work needed to implement the direct observation method to the estimation of the DMI in heterogeneous vegetation without reducing the accuracy of the results.

## Limitations

The M1 must first be used to build or expand the BC database in areas where M2 is implemented for the first time to improve precision. In addition, while a reliable estimation of the DMI is obtained, detailed information regarding the plants species selected is reduced.

## Abbreviations

DMI: dry matter intake
BC: bite categories
M1: method 1
M2: method 2
g DM: grams of dry matter
kg: kilograms
cm: centimetres
